# Fucoidan enhances the therapeutic potential of arsenic trioxide and all-trans retinoic acid in acute promyelocytic leukemia, *in vitro* and *in vivo*

**DOI:** 10.18632/oncotarget.10016

**Published:** 2016-06-14

**Authors:** Farzaneh Atashrazm, Ray M. Lowenthal, Joanne L. Dickinson, Adele F. Holloway, Gregory M. Woods

**Affiliations:** ^1^ Menzies Institute for Medical Research, University of Tasmania, Hobart, Tasmania 7000, Australia; ^2^ School of Medicine, University of Tasmania, Hobart, Tasmania 7000, Australia

**Keywords:** APL, fucoidan, synergy, ATRA, arsenic trioxide

## Abstract

The morbidity and mortality associated with current therapies for acute promyelocytic leukemia (APL) remain a significant clinical concern, despite improvements in patient survival. Consequently, the development of adjuvant therapies that increase efficacy while reducing morbidities is important. Reducing the concentration of the toxic drugs in adjuvant therapy has the potential to reduce unwanted side effects. Therefore, this study aimed to determine the synergistic effects of fucoidan, an anti-tumor agent, with current APL therapies.

When the human APL cell line, NB4, was treated *in vitro* with fucoidan plus ATO and ATRA at therapeutic and sub-therapeutic doses, there was an increase in sub-G0/G1 cells, annexin V/PI-positive-apoptotic cells and DNA fragmentation. This reduction in proliferation and increase in apoptosis was accompanied by enhanced myeloid differentiation as indicated by an increased expression of CD11b. This was not observed with the AML cell line Kasumi-1, suggesting specificity for APL.

*In vivo* treatment of APL-bearing mice with fucoidan+ATRA or fucoidan+ATO delayed tumor growth, induced differentiation and increased tumor volume doubling time. The differentiated APL cells derived from the excised tumor mass exhibited decreased CD44 expression in fucoidan+ATRA treated mice. This could translate to decreased cell migration in APL patients.

Our findings provide evidence supporting the use of fucoidan as an adjuvant therapeutic agent in the treatment of APL.

## INTRODUCTION

Fucoidan, a natural substance derived from marine algae, has immunomodulatory and cytotoxic activities and has been investigated as a potential anti-cancer agent [1, 2]. It has been shown to induce apoptosis in different tumor cells *in vitro* and *in vivo* [3]. Studies have also reported the role of fucoidan in modulation of the immune system through activation of innate and adaptive immune cells and cytokine production [3, 4]. The cytotoxic and immunomodulatory effects have led to the proposal of fucoidan as a putative adjuvant therapy in combination with standard therapies. Synergistic effects of fucoidan with standard anti-cancer components have been reported. Fucoidan plus resveratrol has been shown to decrease the colony growth of the HCT 116 colon cancer cell line by 60% compared to 34% and 27% in resveratrol alone or fucoidan alone, respectively [5]. In a clinical trial, administration of oral fucoidan combined with standard chemotherapy, significantly decreased general fatigue in patients with colorectal cancer compared to those who only received standard chemotherapy. In addition, over a 15-month follow-up, the survival rate of patients who received fucoidan was longer than that of the control group [6]. Mechanisms underlying the anti-cancer activity of fucoidan, as well as other information such as route and dose of administration, and its side effects have been previously reviewed [7].

Acute promyelocytic leukemia (APL) is one of the more aggressive types of acute myeloid leukemia (AML), characterized by accumulation of abnormal promyelocytes with the chromosomal translocation t(15;17) [8]. In recent years there have been considerable improvements in efficacy of therapy, which has been attributed to the introduction of the combination therapy, all-trans retinoic acid (ATRA) and anthracycline [9, 10]. The combination of ATRA and arsenic trioxide (ATO) has also proven effective, particularly for treatment of high risk and relapsed disease [11], however significant clinical challenges remain [12, 13].

ATO is believed to induce apoptosis in a caspase-independent mechanism through increased accumulation of reactive oxygen species (ROS), mitochondrial membrane potential loss, translocation of apoptosis-inducing factor from mitochondria to the nucleus and finally cleavage of PARP-1 (poly (ADP-ribose) polymerase-1), a key enzyme involved in DNA repair [14]. The cleaved PARP-1 fails to repair DNA damage, resulting in apoptosis. In a previous study, we demonstrated that fucoidan induced apoptosis through a caspase-dependent mechanism and further inactivation of PARP-1 in acute promyelocytic leukemia NB4 and HL60 cell lines [15]. Both fucoidan and ATO result in cleavage of PARP-1 but through two different pathways, therefore we hypothesized that the combination of these two agents could synergistically enhance apoptosis in APL cells.

While ATRA provides an effective differentiation-based therapy for APL, the prolonged administration of high doses of ATRA can be associated with the emergence of resistance [16]. Moreover it can cause differentiation syndrome; a potentially fatal complication which occurs in approximately a quarter of APL patients [17]. Some reports show that efficiency of ATRA induced myeloid differentiation may be diminished as a result of decreased retinoic acid receptor alpha (RARα) [18]. Therefore, it is of interest to develop complementary treatment strategies which increase the sensitivity of myeloid cells to ATRA action. Here, we hypothesized that the addition of fucoidan as adjuvant to ATRA might enhance myeloid cell differentiation induced by this agent.

In the present study, the synergistic effects of fucoidan with ATO on ATO-induced apoptosis and with ATRA+ATO on myeloid differentiation were investigated in acute promyelocytic leukemia cells using both *in vitro* and *in vivo* models. We postulated that lower concentrations of ATRA and ATO could attenuate their undesirable side effects. Therefore, the synergistic effects of fucoidan with ATRA and ATO were investigated at sub-pharmacological doses of these agents. In addition, as studies have reported that the adhesion molecule CD44 may play a role in migration and extra-medullary infiltration of leukemic cells [19], we examined the effect of combined fucoidan and ATRA on expression of CD44 in APL cells *in vivo*.

## RESULTS

### Fucoidan synergizes ATO-mediated apoptosis in APL cells

**Figure 1:**
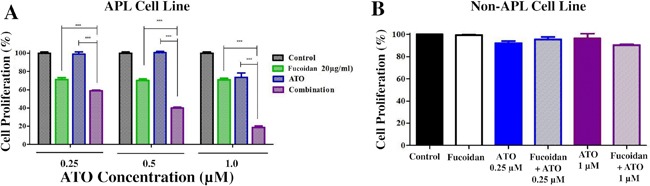
WST-8 cell proliferation assay **A.** NB4 cells were treated with increasing doses of ATO with or without fucoidan (20μg/ml) and cell proliferation was assessed after 48 hours. Tumor cell viability significantly decreased when fucoidan was combined with ATO at both low and therapeutic doses. Mean ± SEM values of three replicates are shown. Statistical significance was determined by two-way ANOVA, followed by Bonferroni post-test (***: p<0.001). **B.** Non-APL Kasumi cell line was co-treated with ATO and fucoidan and cell proliferation was assessed after 96 hours. Neither alone nor combination of agents affect the non-APL cells viability. Mean ± SEM values of three replicates are shown.

The acute promyelocytic leukemia NB4 cell line was treated with 20 μg/ml fucoidan and increasing concentrations of ATO for 48 hours and cell proliferation was assessed. NB4 cell proliferation was significantly decreased when cells were co-treated with fucoidan and ATO, compared to single treatment with either agent (Figure 1A). Decreased proliferation was observed at both low (0.25, 0.5 μM) and clinical doses of ATO (1 μM). Identical results were obtained with 10 μg/ml fucoidin (data not shown). To examine whether the ATO activity observed was specific to APL cells, the Kasumi-1 AML cell line was similarly treated with ATO and fucoidan. Neither combined ATO/fucoidan nor single treatment with fucoidan and ATO decreased the proliferation of Kasumi-1 cells, and the percentage of viable cells in all groups remained at greater than 80% for up to 96 hours (Figure 1B).

**Figure 2:**
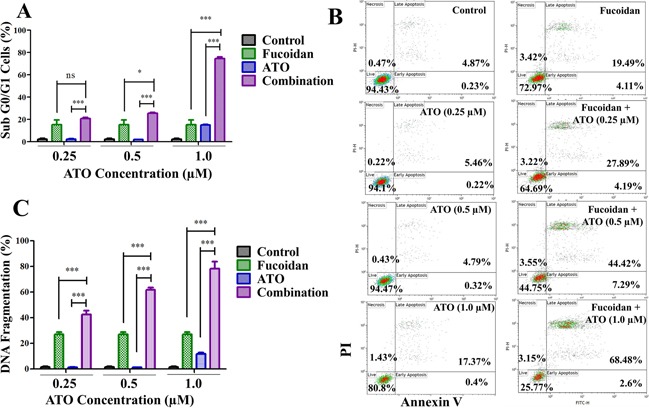
Apoptosis assays NB4 cells were treated with increasing doses of ATO with or without fucoidan (20 μg/ml) and various assays conducted after 48 hours. **A.** DNA content was analyzed and sub G0/G1 population representing dead cells was calculated. Mean ± SEM values of three replicates are shown. Statistical significance was determined by two-way ANOVA, followed by Bonferroni post-test. **B.** Representative annexin V/PI apoptosis assay. The amount of apoptotic cells (positive Annexin V) increased when fucoidan was combined with ATO at both low and high doses. Mean ± SEM values of three replicates are shown. **C.** DNA fragmentation. The amount of apoptotic cells with fragmented DNA significantly increased when fucoidan was combined with ATO at both low and high doses. Mean ± SEM values of three replicates are shown. Statistical significance was determined by two-way ANOVA, followed by Bonferroni post-test (***: p<0.001, **: p<0.01, *: p< 0.05).

To investigate the combined effect of treatment with fucoidan and ATO on cell cycle, the propidium iodide assay was conducted by flow cytometry. The sub-G0/G1 population, representing dead cells, significantly increased when cells were treated with fucoidan+ATO compared to ATO alone at low and therapeutic doses (p<0.001) (Figure 2A). Table 1 shows the distribution of all cell cycle phases. As shown, the combination of ATO with fucoidan decreased G0/G1, S and mitotic phase populations compared to the single ATO.

**Table 1:** Cell cycle phases' distribution in NB4 cells treated with fucoidan, ATO or in combination

		Sub G0/G1	G0/G1	S	Mitosis
**ATO 0.25 μM**	Control	1.93 ± 0.3	42.46 ± 1.39	22.34 ± 0.06	31.67 ± 1.34
	Fucoidan	18.34 ± 1.75	28.66 ± 1.42	22.38 ± 2.7	29.18 ± 3.38
	ATO	2.73 ± 1.08	45.10 ± 0.54	20.22 ± 0.41	30.39 ± 0.87
	Combination	20.69 ± 1.54	31.30 ± 0.09	25.8 ± 2.68	19.44 ± 2.04
**ATO 0.5 μM**	Control	1.93 ± 0.3	42.46 ± 1.39	22.34 ± 0.06	31.67 ± 1.34
	Fucoidan	18.34 ± 1.75	28.66 ± 1.42	22.38 ± 2.7	29.18 ± 3.38
	ATO	2.39 ± 1.08	46.10 ± 0.54	20.22 ± 0.41	29.39 ± 0.87
	Combination	25.57 ± 0.86	38.42 ± 1.38	15.20 ± 0.34	18.14 ± 1.66
**ATO 1.0 μM**	Control	1.93 ± 0.3	42.46 ± 1.39	22.34 ± 0.06	31.67 ± 1.34
	Fucoidan	18.34 ± 1.75	28.66 ± 1.42	22.38 ± 2.7	29.18 ± 3.38
	ATO	15.13 ± 0.4	39.81 ± 3.82	16.56 ± 0.8	25.50 ± 2.11
	Combination	74.51 ± 2.26	7.29 ± 1.36	9.28 ± 2.28	6.76 ± 0.7

NB4 cells were treated with with low and therapeutic doses of ATO with or without fucoidan (20 μg/ml) and cell cycle was analyzed after 48 hours. Data represents Mean±SEM of three replicates.

To determine if cell death was due to apoptosis, the annexin V/PI assay was employed. At low doses of ATO, the percentage of apoptotic cells (annexin V positive cells) increased from approximately 6% (0.25 μM ATO only) to 32% (fucoidan+0.25 μM ATO) and from 5% (0.5 μM ATO only) to 52% (fucoidan+0.5 μM ATO) (p<0.001) (Figure 2B). At the therapeutic dose of 1 μM ATO, the percentage of apoptotic cells increased from approximately 18% (ATO only) to 71% (fucoidan+ATO) (p <0.001).

The TUNEL assay was employed to measure the amount of DNA fragmentation, a hallmark of apoptosis. In line with the annexin V/PI assay data, co-treatment of fucoidan with ATO significantly enhanced apoptosis compared to treatment with ATO alone (p<0.001). As shown in Figure 2C, the mean percentage of cells with fragmented DNA increased to 42.5% (fucoidan+0.25 μM ATO), 60% (fucoidan+0.5 μM ATO), and 79% (fucoidan+1 μM ATO) compared to 1.5%, 1% and 12%, respectively, for the same doses of ATO alone.

### Fucoidan enhances ATRA-induced differentiation in APL cells

**Figure 3:**
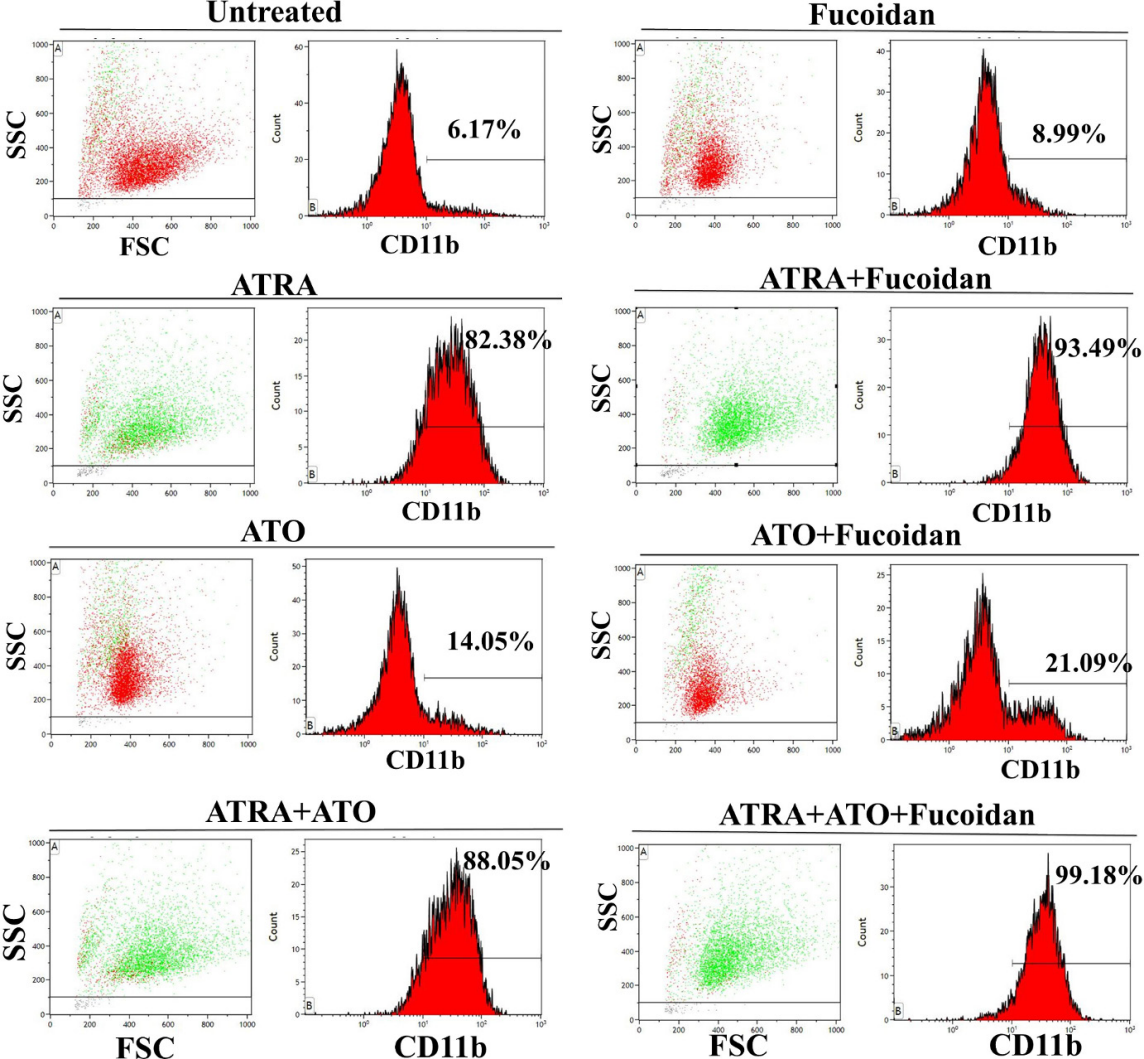
Representative cell differentiation assay NB4 cells were treated with low concentration of fucoidan alone (5 μg/ml), low dose of ATRA alone (0.5 μM), low dose of ATO alone (0.5 μM) or in combination and incubated for 5 days. Myeloid differentiation was analyzed by flow cytometry using CD11b expression. Representative percentages display CD11b^high^ expressing population indicative of differentiated cells in each treated group. In plot histograms, green population demonstrates differentiated population (CD11b^high^) while red population represents non/low-differentiated cells (CD11b^neg/low^).

**Figure 4:**
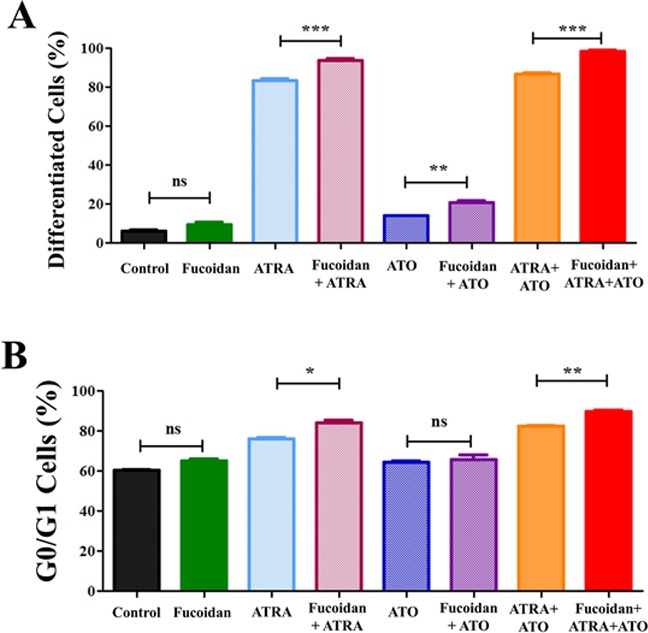
Cell differentiation assays NB4 cells were treated with low concentrations of fucoidan alone (5 μg/ml), low dose of ATRA alone (0.5 μM), low doses of ATO alone (0.5 μM) or their combination. Treated cells were incubated for 5 days and differentiation was analyzed by flow cytometry using CD11b expression and cell cycle assays. **A.** CD11b^high^ expressing population indicative of differentiated cells. As shown, fucoidan promotes ATRA and ATO induced differentiation in APL cells. Mean ± SEM values of three replicates are shown. Statistical significance was determined by ANOVA, followed by Tukey's multiple comparison test. **B.** DNA content analysis. G0/G1 phase arrest was promoted when fucoidan was combined with ATRA and ATRA+ATO NB4. Mean ± SEM values of three replicates are shown. Statistical significance was determined by ANOVA, followed by Tukey's multiple comparison test (***: p<0.001, **: p<0.01, *: p<0.05).

NB4 cells were treated with either fucoidan, low doses of ATRA or ATO alone or in combinations for 5 days. Fucoidan has cytotoxic effects on NB4 cells, therefore a very low dose of fucoidan (5 μg/ml) was selected for this assay to minimize cell death over the 5-day incubation time. Myeloid differentiation was then measured by determining the expression level of CD11b (identified by mean fluorescence intensity). Non-differentiated or low-differentiated cells were identified by CD11b^neg/low^ expression and excluded, whereas differentiated cells were identified by CD11b^high^ expression. The CD11b^high^ cells increased when NB4 cells were co-cultured with fucoidan plus ATRA, ATO or ATRA+ATO compared to ATRA, ATO or ATRA+ATO alone (A representative experiment shown in Figure 3). Single treatment with 0.5 μM ATRA increased the differentiated cells from approximately 6% to 83%, while addition of fucoidan to ATRA increased the differentiated cells to 94%. Low dose ATO similarly increased the differentiated cells from 6% to 14% while fucoidan+ATO enhanced the differentiated cells percentage to 20%. Finally, APL cells treated with all three reagents in combination increased the differentiated cells to 99.5% after the 5-day incubation period, compared to 87% when cells were co-treated with ATRA+ATO (Figure 4A).

At the very low doses of fucoidan used, the amount of sub-G0/G1 dead cell population did not change in treated cells compared to the untreated cells (Table 2). Since cells undergoing differentiation exit the cell cycle during the dividing phase, we analyzed G0/G1 arrest and compared the G0/G1 population within treated groups. In line with CD11b expression data, a significant increase in G0/G1 population was observed in cells co-treated with ATRA+fucoidan compared to ATRA only and in cells co-treated with ATRA+ATO+fucoidan compared to ATRA+ATO (Figure 4B). Table 2 shows detailed results for cell cycle phases of viable cells.

**Table 2:** Cell cycle phases' distribution in NB4 cells treated with fucoidan, ATRA, ATO or in combinations

		G0/G1	S	Mitosis
**Agents alone**	Control	60.64 ± 0.41	20.65 ± 1.1	18.07 ± 0.89
	Fucoidan	65.1 ± 1.54	20.77 ± 1.44	13.3 ± 0.1
	ATRA	76.01 ± 1.05	8.53 ± 1.0	15.12 ± 0.08
	ATO	64.4 ± 0.95	18.29 ± 1.29	16.64 ± 0.31
**Combination**	ATRA+fucoidan	84.28 ± 1.81	5.24 ± 0.21	10.24 ± 1.61
	ATO+fucoidan	66.01 ± 2.95	21.94 ± 1.77	11.38 ± 1.02
	ATRA + ATO	82.44 ± 0.53	6.78 ± 0.06	10.58 ± 0.65
	ATRA+ATO+fucoidan	89.86 ± 1.11	4.42 ± 0.94	5.41 ± 0.16

NB4 cells were treated with ATRA (0.5 μM) and ATO (0.5 μM) with or without fucoidan (5 μg/ml) and cell cycle was analyzed after 5 days. Data represents Mean±SEM of three replicates.

### Co-treatment of fucoidan with ATO and ATRA significantly delays the growth of APL in mice

To evaluate the possible synergy of these agents *in vivo*, we established a subcutaneous xenograft APL mass in nude mice and assessed the agents' effects on the regression of the tumor. The experiments for synergy of fucoidan+ATO (4 groups of 7) and fucoidan+ATRA (4 groups of 8) were performed separately. Treatment commenced as soon as a visible tumor mass (4~10 mm^3^) appeared, which was after 8-14 days in all mice. During the treatment, none of the mice showed any side effects (such as weight loss and/or behavioral changes). Mice were sacrificed when tumor volume reached 1000 mm^3^ (considered as end point).

#### Fucoidan plus ATO

**Figure 5:**
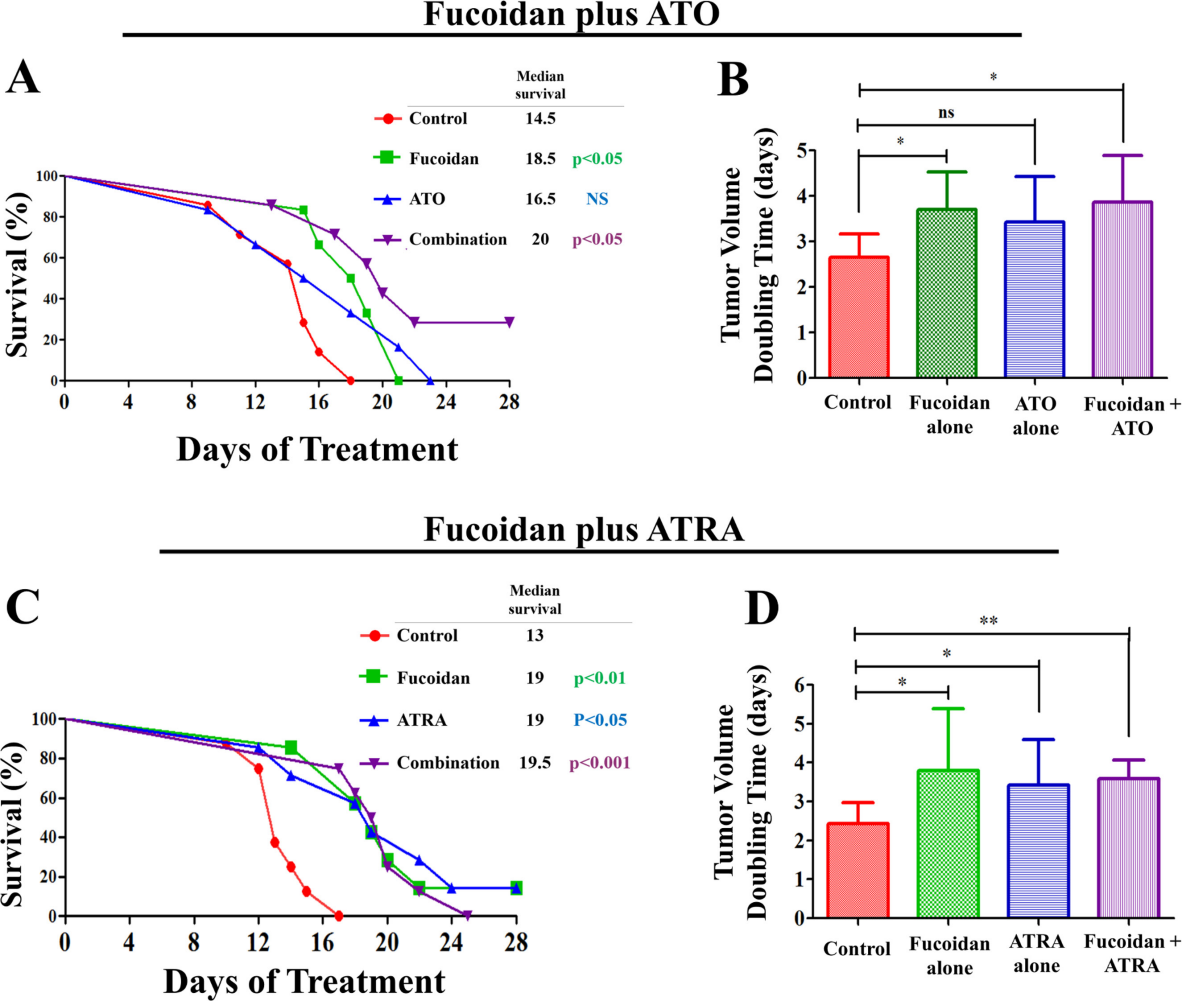
Survival analysis and tumor volume doubling time in APL bearing mice The APL bearing mice were treated with fucoidan, ATO and ATRA. **A.** The Kaplan-Meier survival curve for mice treated with fucoidan, ATO and their combination compared to the control group for over 28 days of treatment. Mice were sacrificed when tumor volume reached 1000mm^3^ **B.** Tumor volume doubling time in mice treated with fucoidan, ATO and their combination compared to the control group (n=7/group) (mean ± SD). **C.** The Kaplan-Meier survival curve for mice treated with fucoidan, ATRA and their combination compared to the control group for over 28 days of treatment. **D.** Tumor volume doubling time indicative of tumor aggressiveness in mice treated with fucoidan, ATRA and their combination compared to the control group (n=8/group) (mean ± SD). Statistical significance was determined by ANOVA, followed by Tukey's multiple comparison test (**: p<0.01, *: p<0.05). The inserted ‘table’ represents the median survival and the statistical significance is compared to the control.

Of the 7 mice treated with fucoidan+ATO, 2 mice completed the full 28-days treatment. All mice in the other groups did not complete the full 28-days treatment and were sacrificed as per animal ethics guidelines prior to 28 days as tumor volume reached 1000 mm^3^. Treatment with “fucoidan only” and “fucoidan+ATO” significantly repressed the development of the tumor mass and the tumor in these animals reached the maximum end point at a longer duration than the control group (p<0.05). The median survival was 14.5, 18.5, 16.5 and 20 days in control, fucoidan alone, ATO alone and combination groups, respectively (Figure 5A). Despite higher median survival in the combination group, the difference between treated groups was not statistically significant. The tumor aggressiveness evaluated by tumor volume decreased in treated groups compared to the control group. The mean tumor volume doubling time was 2.72, 3.70, 3.42 and 3.86 days in control, fucoidan, ATO and combination groups, respectively (Figure 5B).

#### Fucoidan plus ATRA

In the analysis of anti-tumor effects of ATRA plus fucoidan, one animal in the fucoidan alone group and one animal in the ATRA alone group completed the full 28-days treatment. The remaining mice did not complete the full 28-days treatment and were sacrificed as per animal ethics guidelines prior to 28 days as tumor volume reached 1000 mm^3^. A significant regression of the APL tumor was seen in all three treated groups compared with the control group. The median survival was 13, 19, 19 and 19.5 days in control, fucoidan alone, ATRA alone and combination groups, respectively (Figure 5C).

Cell growth appeared to slow as indicated by mean tumor volume doubling time, which was 2.43, 3.92, 3.42 and 3.53 days in control, fucoidan, ATRA and combination groups, respectively (Figure 5D). The increase in tumor volume doubling time in treated groups was significantly larger than that of the control group, but did not significantly differ from each other.

### Fucoidan significantly increases the differentiation of APL cells *in vivo*

At the end of the *in vivo* study of the combinatory effect of fucoidan plus ATRA, the tumor mass was removed and NB4 cell differentiation was assessed by CD11b expression. Since almost all NB4 cells highly express CD44, tumor cells were identified by CD44 expression.

**Figure 6:**
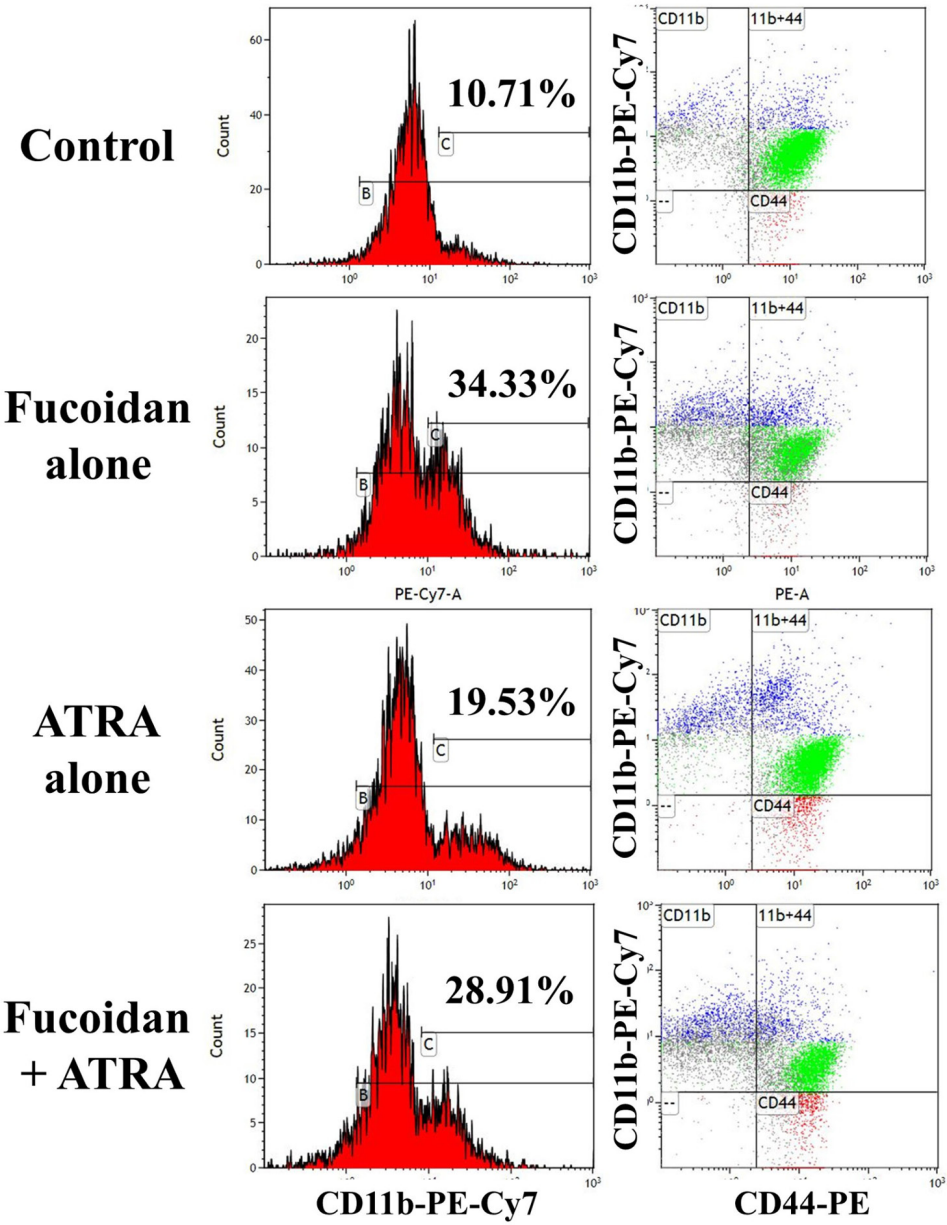
Representative flow cytometry analysis of differentiation in NB4 cells derived from mice tumor mass The APL bearing mice were treated with fucoidan, ATRA or their combination and myeloid differentiation in tumor mass was calculated using CD11b expression. The percentage of differentiated cells with CD11b^high^ was calculated (left graphs). The right graphs demonstrate the expression of CD11b and CD44. Blue population: differentiated cells (CD11b^high^CD44^+^), green population: non/low differentiated cells (CD11b^neg/low^CD44^+^). Histograms show the flow cytometry results in one representative mouse in each group.

**Figure 7:**
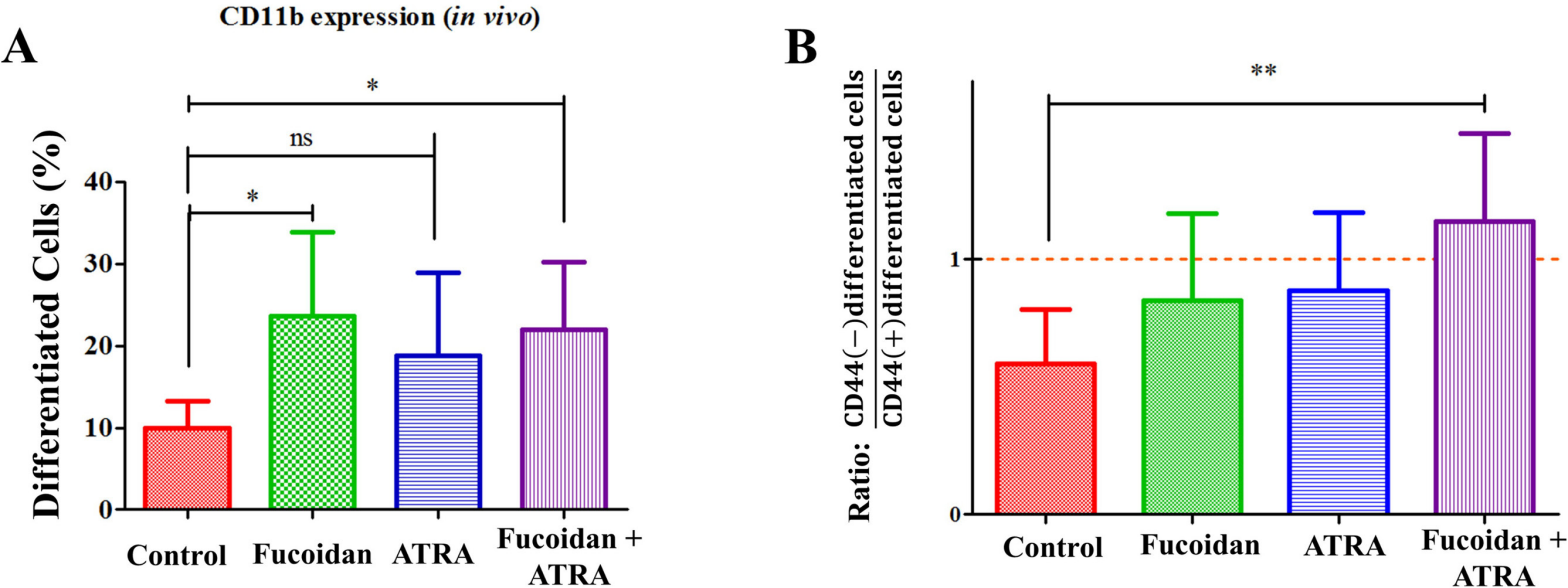
**A.** The myeloid differentiation in tumor mass obtained from mice. Differentiation was measured using CD11b expression in tumor mass obtained from mice treated with fucoidan, ATRA and combination compared to control group. Each column represents the mean ± SD values of CD11b^high^ cells in each experimental group (n=8/group). Statistical significance was determined by ANOVA, followed by Tukey's multiple comparison test (*: p<0.05). **B.** The CD44^−^/CD11b+ Cells to CD44^+^/CD11b+ Cells ratio in tumor mass obtained from mice. The ratio between CD44^−^ to CD44^+^ differentiated cells in tumor mass obtained from mice treated with fucoidan, ATRA and combination compared to the control group. Statistical significance was determined by ANOVA, followed by Tukey's multiple comparison test (**: p<0.01). Each column represents the mean ratio of CD44^−^ to CD44^+^ differentiated cells with error bars the SD.

A significant increase in differentiation was observed in the tumor mass treated with fucoidan alone and fucoidan+ATRA (p<0.05) (A representative experiment is shown in Figure 6). The mean values of differentiated cells were approximately 10%, 24%, 19% and 22% in control, fucoidan, ATRA and combination groups, respectively (Figure 7A). There was no significant difference between treated groups with each other.

Furthermore, a different immunophenotypic pattern in expression of CD44 and CD11b was observed in different treated groups (blue population in Figure 6). Evaluating the expression of CD44 on differentiated cells, the ratio of CD11b^high^CD44^−^ cells to CD11b^high^CD44^+^ cells was calculated. This ratio was 0.58, 0.83, 0.87 and 1.14 in control, fucoidan alone, ATRA alone and combination groups, respectively, indicating decreased expression of CD44 in mice treated with fucoidan+ATRA (Figure 7B).

## DISCUSSION

Severe toxicity is frequently observed in newly diagnosed APL patients treated with traditional chemotherapeutic agents. Studies and trials have investigated whether the combination of ATRA+ATO with minimal or no chemotherapy can replace standard therapy [12]. Hematological remission is observed in 80-90% of refractory and relapsed patients treated with ATRA+ATO [20], however the use of this combination in newly diagnosed and high-risk patients remains clinically challenging and can induce serious complications such as hyperleukocytosis and differentiation syndrome [21, 22]. Therefore, the use of adjuvants to increase the efficiency of ATO+ATRA while decreasing the risk of side effects has gained interest.

Fucoidan has as anti-tumor and immunomodulatory effects and its low toxicity makes it beneficial for a potential adjuvant therapy [7]. Zhang *et al.* reported that combining fucoidan with standard chemotherapeutic agents increased apoptosis in breast cancer cell lines, potentially through the induction of oxidative stress [23]. Zuo and colleagues showed that chemotherapy induced intestinal damage was significantly reduced when mice also received fucoidin. This was most likely by improving intestinal immune system function [24]. Previously, we have shown that fucoidan selectively inhibits cell growth of APL cells *in vitro* [15]. The present study determined if fucoidan can enhance the effectiveness of standard treatment of APL.

At clinically achievable concentrations of 0.5-2 μM, ATO induces apoptosis in malignant promyelocytes. Furthermore, at clinical doses of 1 μM and 0.1-0.5 μM, ATRA and ATO respectively stimulate partial or terminal differentiation of the accumulated abnormal promyelocytes [25, 26]. Here, *in vitro*, we have shown that combining fucoidan with ATO and ATRA enhanced apoptosis and differentiation at both clinically-relevant and sub-therapeutic doses of ATRA and ATO. It should be noted that we used a lower dose of fucoidan (20 μg/ml) than previously used [15] and even this low dose significantly decreased cell proliferation.

Few studies have examined the combinatory activity of natural derived compounds with ATO in treatment of APL. Lu *et al*. reported that combination of a crude methanolic extract of *Mucana macrocapra* with ATO suppressed proliferation of the APL cell line HL60. It also increased apoptosis through accumulation of ROS [27]. In a recent investigation, combination of Icarrin, a natural flavonoid derived from several plants, with ATO significantly increased NB4 cell apoptosis through excessive cellular oxidative stress [28]. The aforementioned studies used high doses of ATO; from 2.5 to 12 μM, which are considerably higher than clinical doses. To reduce side effects, we propose that lower doses of ATO could be used in combination with fucoidan. By treating APL cells with sub-clinical doses of ATO and fucoidan, the efficacy of ATO in induction of apoptosis was significantly increased. This would have the benefit of minimizing the toxicities of ATO.

We and others have shown that fucoidan decreases the activity of AKT [29, 30]. AKT, a serine/threonine kinase, is a key molecule in the PI3K/AKT signaling pathway which inhibits apoptosis and is frequently increased in various tumors [31]. Further there are several reports demonstrating that ATO triggers apoptosis via inhibition of AKT [32, 33]. As fucoidan and ATO appear to trigger the same pathway, it is possible that the synergistic activity is due to a double hit of the AKT pathways. In addition, apoptosis mediated by ATO is caspase independent [15, 34, 35], while fucoidan cytotoxicity action on NB4 cells is caspase-dependent [15, 36, 37]. Although fucoidan and ATO recruit different pathways, both agents eventually induce cleavage of PARP-1, the main enzyme in DNA repair [14]. This may explain the increased DNA fragmentation by fucoidan plus ATO compared to ATO alone.

Failure to differentiate is a feature of the abnormal promyelocytes in APL [38]. *In vitro*, we found that co-stimulation of NB4 cells with fucoidan and ATRA+ATO resulted in synergistic induction of myeloid differentiation characterized by increased expression of CD11b and G0/G1 arrest. When fucoidan was combined with ATRA+ATO, almost all cells underwent differentiation. In contrast, when cells were treated with ATRA alone or ATRA+ATO, a proportion of cells remained undifferentiated. During chemotherapy, the drug-resistant cell population is the main source of cancer recurrence in patients. Our findings suggest that the number of resistant cells in response to ATRA could be limited in patients by addition of fucoidan to the standard APL regimen.

ATRA and ATO induce differentiation through the degradation of the oncoprotein PML-RARα, the main barrier of differentiation in APL cells [39]. Previous studies on fucoidan's anti-cancer effects have shown that fucoidan mostly regulates the protein levels rather than mRNA levels. Hence it will be worthwhile to investigate the effects of fucoidan on the expression of PML-RARα fusion protein as it might alter the expression of the fusion protein or enhance its degradation. Furthermore, multiple signaling pathways have been implicated for the action of both fucoidan and ATRA. For instance, inactivation of JNK and ERK, and inhibition of transcription factor NFκB, which normally promotes cell proliferation, have been reported for ATRA-induced differentiation in NB4 cells [40]. Similar activities have been reported for fucoidan [30, 41]. The double modulation of signaling pathways and other related molecules by these two agents could contribute to the observed synergy. Further investigations including gene transcription profiling are required for better understanding of the underlying mechanisms.

Despite many *in vitro* studies investigating the synergistic activity of fucoidan with anti-cancer drugs, *in vivo* studies have been limited. Alekseyenko and colleagues reported that in C57Bl/6 mice fucoidan significantly potentiated the anti-metastatic, but not anti-tumor activity of the chemotherapeutic agent cyclophosphamide [42]. In a clinical trial, fucoidan similarly did not affect the efficacy of standard chemotherapeutic agents in patients with colorectal cancer, but reduced the toxicities of chemotherapy [6]. Our studies of the synergistic effects of fucoidan with ATO and ATRA *in vivo* showed an enhanced lifespan and delayed tumor growth when mice were treated with fucoidan+ATO and fucoidan+ATRA.

It has been well established that high doses of ATO and ATRA can cause side effects. In a study by Jing *et al*., 8 μg/g b.w. ATO and 10 μg/g ATRA increased the lifespan of mice bearing NB4 cells. But combination of these agents was toxic with weight loss and early therapy-associated death [26]. Lallemand-Breitenbach *et al*. also reported that 10 μg/g b.w. led to many early deaths. Hence, they used a reduced dose of 5 μg/g b.w. ATO in mice bearing APL [43]. In our study, with the assumption that the lower concentrations could attenuate the side effects of these drugs, we reduced the dose even further; 2.5 μg/g b.w. ATO and 1.5 μg/g b.w ATRA, and observed no toxicity when they were used alone or in combination with fucoidan. We believe that the lower cytotoxicity activity observed in ATRA+fucoidan study than ATO+fucoidan study could be due to the ultra-low dose ATRA used. Despite the very low doses utilized, the efficacy of combined treatment was still higher than that of standard APL treatment.

To address the effects of fucoidan on APL cell differentiation *in vivo*, we lastly measured the differentiation of NB4 cells obtained from the tumor mass of mice treated with fucoidan and ATRA. In contrast with our *in vitro* findings, in which fucoidan alone had little effects on NB4 cell differentiation, *in vivo,* fucoidan alone induced more differentiation in NB4 cells compared to ATRA alone. Granulocytic differentiation includes a highly regulated process which occurs upon stimulation of several cytokines [44]. The microenvironment and cellular cross talk are other critical factors that can influence the maturation and differentiation process. As fucoidan has been shown to change the production of various cytokines *in vivo* [4], we believe the different results between fucoidan's action *in vivo* and *in vitro* could be explained by alteration of cytokines that have regulatory effects on myeloid maturation. Furthermore, it has been reported that fucoidan has a synergistic effect with granulocyte colony stimulating factor (G-CSF) to alter leukocyte trafficking and mobilization [45]. Changes in the pattern of circulatory blood cells combined with the altered stimulation of cytokine production by immune cells could lead to a different microenvironment around engrafted NB4 cells compared to *in-vitro*-cultured NB4 cells. G-CSF is a major cytokine which increases granulocytic differentiation [46]. Synergy of fucoidan with G-CSF in induction of differentiation is another hypothesis which needs to be investigated in future studies.

In our study, the combined fucoidan and ATRA treatment of mice bearing APL tumors not only caused myeloid differentiation, but reduced CD44 expression. This was not observed in those mice treated with ATRA or fucoidan alone. CD44 is an adhesion molecule involved in cell proliferation, differentiation, migration and angiogenesis [47]. It is well documented that up-regulation of CD44 on cancer cells is correlated with poor prognosis as it can enhance cell migration and metastasis [48]. Inhibition of CD44 using specific ligands has been shown to reduce trans-endothelial migration of AML cells by 78% [49]. Sun *et al*. have also reported that pre-treatment of acute promyelocytic leukemia NB4 cells with anti-CD44 antibody decreased both the cell-cell adhesion rate and cell migration by 50% compared to the control group [19]. As mentioned above, development of the differentiation syndrome caused by ATRA and ATO is one of the main clinical challenges in treatment of APL, at which the accumulated differentiated young neutrophils infiltrate into various body sites, particularly lungs, and cause potentially fatal complications [50]. We suggest that the increase of differentiated cells with low or no expression of CD44 in mice treated with ATRA plus fucoidan adds further evidence that it may decrease the migration of these cells. However, further analysis including developing murine models of differentiation syndrome following treatment with ATRA plus fucoidan is needed.

In conclusion, our findings suggest that the use of fucoidan as a supplementary agent to the APL standard treatment may represent a promising new strategy for management of APL.

## MATERIALS AND METHODS

### Cell culture and cell treatment

The t(15;17) translocation positive human acute promyelocytic leukemia NB4 cell line [51] was cultured in RPMI 1640 medium containing L-glutamine (Gibco®, Life Technologies Australia, Melbourne, Australia) supplemented with 10% fetal calf serum, 100 U/ml penicillin, 100 μg/ml streptomycin (Sigma-Aldrich Australia, Castle Hill, Australia) at 37°C and 5% CO_2_. The non-APL Kasumi-1 myeloid leukemia cell line (ATCC (^®^CRL-2724™)) [52] was selected for comparison and cultured in RPMI 1640 medium containing L-glutamine supplemented with 20% fetal calf serum at 37°C and 5% CO_2_.

Fucoidan derived from *Fucus vesiculosus* (Sigma-Aldrich Australia, Castle Hill, Australia; product number: F-5631) was dissolved in PBS and filtered (0.21-μm Millipore syringe filter). Endotoxin content was measured using chromogenic LAL endpoint assay (GenScript, *Piscataway,* NJ, USA) to be less than 0.1 EU/ml, which is considered as non-pyrogenic.

For *in vitro* experiments, a stock solution of 10 mM arsenic trioxide (ATO) (Sigma-Aldrich Australia, Castle Hill, Australia) was prepared in 1.65 M NaOH and a stock solution of 10 mM all-trans retinoic acid (ATRA) (Sigma-Aldrich Australia, Castle Hill, Australia) was prepared in absolute ethanol.

Cytotoxicity assays in our previous published study showed that 50-100 μg/mL fucoidan induced apoptosis in almost all NB4 cells. Due to the cytotoxic effect of fucoidan in NB4 cells, very low concentration of fucoidan was selected in this study than previously used [15]. The lower doses of fucoidan allowed us to identify the effect of fucoidan on ATO-induced apoptosis. For apoptosis analysis, NB4 cells were treated with fucoidan (20 μg/ml) alone, and increasing concentrations of ATO (0.25, 0.5, 1 μM) alone and in combination and were incubated for 48 hours as indicated. Kasumi-1 cells were treated similarly for up to 96 hours. For differentiation analysis, NB4 cells were treated with low concentration of fucoidan alone (5 μg/ml), low dose of ATRA alone (0.5 μM), low dose of ATO alone (0.5 μM) and their combination and were incubated for 120 hours as indicated.

### Cell proliferation and cell differentiation analysis

Cell proliferation was assessed using a colorimetric assay with the tetrazolium salt WST-8 (Sigma-Aldrich Australia, Castle Hill, Australia), according to the manufacturer's instruction. In brief, for the last 4 hours of incubation time, the WST-8 solution was added to the 96-well microculture plate and the absorbance at 450 nm was measured by spectrophotometry (SpectraMax® Plus^384^, Molecular Devices, USA).

Monitoring the myeloid differentiation was performed by detection of the cell surface myeloid differential marker CD11b using flow cytometry and cell cycle assay. Briefly, after 5-days incubation, 1 × 10^6^ cells were incubated with PE-Cy™7 conjugated CD11b antibody (BD Biosciences, San Jose, CA, USA) or matched isotype control antibody (BD Biosciences, San Jose, CA, USA) at 4°C for 30 minutes. The expression of CD11b was analyzed by FACSCanto™II flow cytometer (BD Biosciences, San Jose, CA, USA) and flow cytometry data was analyzed using Kaluza® 1.2 Software (Beckman Coulter Inc., Brea, CA, USA.). The differentiated cells were distinguished using median fluorescence intensity (MFI) and the percentage of cells with high MFI was recorded.

### Cell cycle assay

Evaluation of the cell cycle distribution was performed using propidium iodide (PI) essentially as described previously [53]. Briefly, 1-2 × 10^6^ cells were fixed with 70% ethanol and incubated at −20°C overnight. Cells were washed twice with PBS and re-suspended in PBS containing 40 μg/ml PI (Sigma-Aldrich Australia, Castle Hill, Australia) with 100 μg/ml DNAse free RNAse A (Qiagen, Melbourne, Australia) and incubated in the dark at room temperature for 15 minutes. The DNA content was analyzed using the FACSCanto™II flow cytometer (BD Biosciences, San Jose, CA, USA) and flow cytometry data was analyzed using Kaluza® 1.2 Software (Beckman Coulter Inc., Brea, CA, USA).

### Cell apoptosis assay

Cells were analyzed for apoptosis using annexin V/PI assay as described previously [54]. In brief, 1-5 × 10^5^ treated cells were washed with PBS and re-suspended in annexin V binding buffer (Abcam Inc., Cambridge, MA, USA). Cells were then stained with annexin V-FITC antibody (Abcam Inc., Cambridge, MA, USA) and PI and incubated at room temperature for 15 minutes in the dark. The early and late apoptotic cells were quantified using the FACSCanto™II flow cytometer (BD Biosciences, San Jose, CA, USA).

Apo-BrdU TUNEL apoptosis assay kit (Invitrogen Ltd, Carlsbad, CA, USA) was used to detect the presence of DNA fragmentation. Briefly, treated cells were washed with PBS, fixed with paraformaldehyde and put on ice for 15 minutes. Cells were washed with PBS and stored in 70% ethanol for 18 hours at −20°C. Cells were then washed twice with PBS and re-suspended in a DNA-labelling solution containing terminal deoxynucleotidyl transferase (TdT) and 5-Bromo-2′-deoxyuridine 5′-triphosphate (BrdUTP) for 60 minutes at 37°C. Following this, cells were rinsed and stained with Alexa Fluor® 488 dye-labeled anti-BrdU antibody. Presence of DNA fragmentation was analyzed by FACSCanto™II flow cytometer (BD Biosciences, San Jose, CA, USA) in parallel with manufacturer-supplied negative and positive control cells.

### *In-vivo* study design

The protocols of the *in-vivo* experiments were approved by the Animal Ethics Committee of University of Tasmania (UTAS), approval number A13940. *In-vivo* experiments were carried out on 5-7 weeks old male Balb/c athymic nu/nu mice from the Department of Animal Service, University of Tasmania. Animals were housed in a pathogen-free environment and provided with food and water *ad libitum*.

Arsenic trioxide solution was prepared by dissolving ATO powder in 1.65 M NaOH followed by diluting in PBS and adjusting the pH to 7.2 for injection. To prepare the ATRA solution for injection, a stock solution of ATRA in DMSO was diluted in sterile PBS.

To examine the anti-tumor effects of fucoidan and its synergy with ATO, 28 mice (n=7/group) were randomly divided into four treatment groups; the control group, the fucoidan group, the ATO group or fucoidan+ATO. A subcutaneous (SC) xenograft tumor was established in mice by injecting 5 × 10^6^ acute promyelocytic leukemia NB4 cells in the right flank of each mouse. Following tumor appearance, treatment was commenced for up to maximum 28 days. Mouse weight and tumor volume was measured daily (Supplementary Table S1). The therapeutic regimens were applied at the following doses: 100 μg/g body weight (b.w.) fucoidan (daily), 2.5 μg/g b.w. ATO (daily) or in combination. All regimens were injected intraperitoneally (IP). The control group was treated with IP injections of sterile PBS. Mouse weight and tumor volume was measured daily by using the formula: volume = (width^2^ × length)/2. Once the transplanted tumor volume reached 1000 mm^3^, this time was considered as the end point and the mice were humanely euthanized with inhalation of CO_2_. Tumor volume doubling time was calculated for each mouse by the algorithm provided by.

To examine the anti-tumor effects of fucoidan and its synergy with ATRA, 32 mice (n=8/group) were randomly divided into four treatment groups; the control group, the fucoidan group, the ATRA group or fucoidan+ATRA group. After tumor establishment (as above), the therapeutic regimens were applied at the following doses: 100 μg/g b.w. fucoidan (daily), or 1.5 μg/g b.w. ATRA (three times a week) or in combination and monitoring was performed as above. The control group was treated with IP injections of vehicle (sterile PBS+DMSO). At the end point (as explained above), mice were sacrificed and the tumor mass was removed. A tumor cell suspension was then prepared and differentiation was analyzed by measuring CD11b expression. Tumor cells were dual stained with human PE-conjugated CD44 antibody and CD11b antibody (BD Biosciences, San Jose, CA, USA).

### Statistical analysis

Data were analyzed for significance using the Student's t-test accompanied by analysis of variance (ANOVA) as indicated in Figure legends using GraphPad Prism 5 Software.

## SUPPLEMENTARY TABLE




