# Supplementary material for: Fucoidan enhances the therapeutic potential of arsenic trioxide and all-trans retinoic acid in acute promyelocytic leukemia, in vitro and in vivo
Source: Oncotarget. 2016 Jun 14;7(29):46028–41. doi: 10.18632/oncotarget.10016 (PMC5216779; doi:10.18632/oncotarget.10016)
Supplement: Supplementary file 1 [file oncotarget-07-46028-s001.pdf]

Fucoidan enhances the therapeutic potential of arsenic trioxide and all-trans retinoic acid in acute promyelocytic leukemia, *in vitro* and *in vivo*

SUPPLEMENTARY TABLE

Supplementary Table S1: Tumor volumes (mm3).

See Supplementary File 1
